# Effects of Eluate Drying on the Chemodiversity of Dissolved Organic Matter Revealed by Ultrahigh-Resolution Mass Spectrometry

**DOI:** 10.1021/acsmeasuresciau.5c00055

**Published:** 2025-06-30

**Authors:** Xinyi Chen, Qing-Long Fu, Ziyong Sun

**Affiliations:** MOE Key Laboratory of Groundwater Quality and Health, School of Environmental Studies, 12564 China University of Geosciences, Wuhan 430074, China

**Keywords:** Natural organic matter, chemodiversity, FT-ICR MS, sample treatment, molecular characteristics

## Abstract

The drying treatment of dissolved organic matter (DOM) eluate was often used to prepare DOM solutions for chemodiversity analysis using Fourier transform ion cyclotron resonance mass spectrometry. However, the effects of drying treatment on the chemodiversity of DOM have not been thoroughly investigated. In this study, vacuum freeze-drying and vacuum centrifuge drying resulted in approximately half and 10% loss of DOM mass loss, respectively. Although the overall values of molecular functional diversity indices and main DOM fractions were insignificantly affected by both drying treatments, the Cl-containing molecules (Cl-OM) and saturated compounds were significantly affected by the drying treatments, particularly for vacuum centrifuge drying. Therefore, the DOM eluate was strongly recommended for the measurement of Fourier transform ion cyclotron resonance mass spectrometry only after dilution by desired folds when the minor DOM fractions, such as Cl-OM and saturated compounds, were of interest. The findings of this study have provided valuable evidence of sample preparation for the accurate elucidation of DOM chemodiversity from various sources.

## Graphical abstract

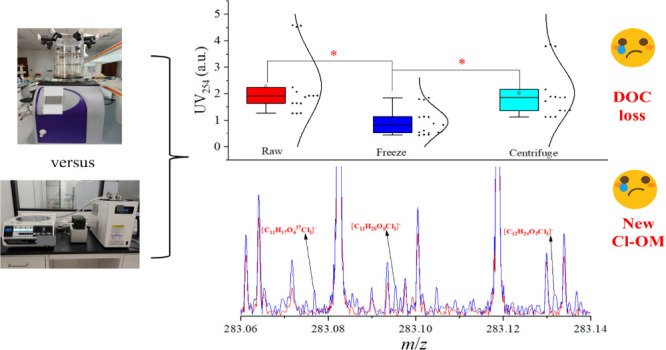

## 1 Introduction

Dissolved organic matter (DOM) is the most reactive carbon pool in aquatic and terrestrial environments. Elucidating DOM molecular chemodiversity is essential to revealing its vital roles in governing the environmental fate and bioavailability of trace metals and organic contaminants, controlling the formation of toxic halogenated disinfection byproducts (DBPs), and affecting the global biogeochemical cycles of carbon and nitrogen.^1–4^ Fourier transform ion cyclotron resonance mass spectrometry (FT-ICR MS) combined with electrospray ionization (ESI) has been the cutting-edge technique for the molecular characterization of DOM since the middle 1990s.^1,5,6^ However, due to the low levels of DOM in natural aquatic systems and the highly sensitive nature of FT-ICR MS to residual inorganic salts, soil phase extraction (SPE) has been the extensively employed method for DOM purification and enrichment.^7,8^

Although some SPE procedures have been proposed to extract DOM,^7,8^ these proposed procedures have often been modified because of various DOM sources and different experimental purposes. Numerous studies have been performed to investigate the effects of sorbents of SPE cartridges, elution conditions, and solvent composition on the chemodiversity of DOM eluate.^9–13^ The SPE-extracted DOM is generally eluted by organic solvents (*e.g*. methanol and acetonitrile), diluted to desired concentrations (50–500 mg-C/L), and then directly injected into an FT-ICR MS instrument for molecular cauterization.^8–10,14–22^ However, in addition to bulk property measurement,^23,24^ the DOM eluate was further subjected to additional drying and redissolving for FT-ICR MS measurement in some specific scenarios.^25–29^ For example, the DOM chromatographically fractionated with organic solvents was dried at 30 °C overnight or under a nitrogen stream to achieve desired analyte concentrations for FT-ICR MS measurement.^27,30^ The elevating analyte concentration during the eluate drying treatment was expected to facilitate the concentration effect of DOM compounds on the product formation of DOM molecules with solvents.^31^ However, the effects of eluate drying on the chemodiversity of DOM with various sources are still poorly revealed by ultrahigh-resolution mass spectrometry, including FT-ICR MS.

The main objective of this study was to compare the chemodiversity of DOM eluate with different sources (*i.e*., tap water, surface water, groundwater, and soil) before and after drying using a vacuum freeze-dryer and vacuum centrifuge dryer at room temperature. The results of this study are expected to provide critical insights into the sample preparation of DOM for FT-ICR MS analysis.

## 2 Materials and Methods

### 2.1 DOM Sampling and Preparation

The tap water was collected in April 2025 from the laboratory of the School of Environmental Studies at China University of Geosciences, Wuhan. Two freshwater samples were collected in April 2025 from a typical urban lake in China (East Lake, 30°19′23.5″N, 114°14′35.6″ E) and the Yangtze River (30°33′41.86″ N, 114°18′13.46″E). The groundwater DOM eluate provided by Ziqi Zhou was collected in March 2024 from Sydney, Australia. A typical forestry soil (5–20 cm) collected from Wuhan Botanical Garden in Hubei Province (30°32′43″N; 114°25′14″E) was used to extract soil DOM with the following procedures: soil mixtures with 10 g of soil and 500 mL of ultrapure water in the glass bottle were shaken at 150 rpm for 12 h and then left to stand for 24 h in the dark at room temperature (25 °C). The soil supernatant and all water samples were filtered through a 0.45 μm membrane and acidified with concentrated HCl (GR grade) to ∼ pH 2. All acidified solutions (∼ 500 to 1500 mL) were gravity-fed through the Oasis HLB cartridges (500 mg/6 cc, Waters, US) preactivated with 120 mL methanol (LC-MS grade), 50 mL ultrapure water, and 20 mL diluted HCl (∼ pH 2). Then, the cartridges were desalted with 20 mL diluted HCl (∼ pH 2) and 20 mL ultrapure water before completely drying with high-purity nitrogen gas (>99.999%). Subsequently, DOM molecules were eluted with 10 mL methanol, which was designated raw DOM in this study. For each DOM sample, an aliquot (2.0 mL) of raw DOM was diluted with an identical volume of ultrapure water, solidified at −80 °C for 3 days (MD-86L456 K, Midea, China), and vacuum freeze-dried at −100 °C under vacuum for 2 days (VirTis Benchtop Pro, USA). The freeze-dried DOM (FD-DOM) was then redissolved into 5.0 mL ultrapure water. Another aliquot (2.0 mL) of each raw DOM was directly dried by vacuum centrifuging at 1300 rpm and 25 °C under vacuum for 180 min using the centrifugal concentrator (CV200, Beijing JM Instrument Co., Ltd., China). The centrifugally dried DOM (CD-DOM) was also redissolved into 5.0 mL ultrapure water. The raw DOM and reconstituted DOM were diluted with ultrapure water for ultraviolet–visible spectrometer measurement. All treatments were performed in triplicate in this study.

### 2.2 FT-ICR MS Measurement

The raw and reconstituted DOM eluate of each triplicate was further diluted to ∼ 10 mg of C/L with 50% methanol before FT-ICR MS analysis with a SolariX 2xR FT-ICR MS instrument equipped with a 7-T superconducting magnet (Bruker, Germany), electrospray ionization operated under the negation ion mode, and quadrupole (2ω) detector at the China University of Geosciences. The mass-to-charge ratio (*m*/z) values were externally calibrated with ion peak clusters of 20 mg/L sodium formate before measurement. The FT-ICR MS spectra were acquired with the instrumental conditions as follows: 120 μL/h direct infusion rate, 100–1,100 *m*/*z* range, 600 scans, 4 Megaword data acquisition size, −3.3 V front and back trap plate voltage, −4.2 kV capillary voltage, and 0.50 s ion accumulation time.

### 2.3 Data Analysis

To improve the reliability of FT-ICR MS results, only peaks that occurred no less than twice among triplicates for each treatment were used in this study.^32,33^ The FT-ICR MS spectra were internally calibrated with known formula homologous series of freshwater DOM and then proceeded to the molecular formulas assignment using our FTMSDeu algorithm under the computation conditions as follows: (1) ion charge sate = −2 to −1; (2) absolute mass error ≤ 0.60 ppm; (3) signal-to-noise ratio (*S*/*N*) ≥ 6 and ≥ 10 for nonhalogenated and halogenated monoisotopic molecular formulas, respectively; (4) 0.3 ≤ (H + Cl + Br)/C ≤ 2.25 and 0< O/C ≤ 1.2 with C ≥ 5; (5) (H + Cl + Br)/C ≤ 4 and 0 ≤ O/C ≤ 1.2 with C ≤ 4; (6) integer double bond equivalent (DBE) ≥ 0; (7) 1≤ ^12^C ≤ 50, ^13^C ≤ 2, ^18^O ≤ 1, ^14^N ≤ 5, ^15^N ≤ 1, ^32^S ≤ 3, ^33^S ≤ 1, ^34^S ≤ 1, P ≤ 1, ^35^Cl ≤ 5, ^37^Cl ≤ 5, ^79^Br ≤ 5, ^81^Br ≤ 5, and (8) 10 ≤ DBE value minus the number of oxygen atom (DBE-O) ≤ 10, and (9) doubly charged formulas were restricted to C, H, O, and N.^34,35^ The molecular parameters, including DBE, modified aromaticity index (AI_mod_), and nominal oxidation state of carbon (NOSC), were calculated with equations reported elsewhere^36,37^ and further weighted based on the intensities of assigned formula in each FT-ICR MS spectrum.^34^ The Bray–Curtis dissimilarity was employed to quantify dissimilarities using the peak intensities of assigned formulas across different FT-ICR MS spectra.^38^ The molecular functional diversity (*DF*) was quantified using the equations described in Text S1.^39–41^ The *DF* reflects the theoretically expected difference value of a given selected property parameter (*m*/*z* value, H/C, AI_mod_, DBE, and NOSC) between any two molecular formulas in a given spectrum. The *DF­(m/z)*, *DF­(H/C)*, *DF­(AI*_*mod*_ *)*, *DF­(DBE)*, and *DF­(NOSC)* indicate the *DF* value based on the molecular parameters of *m*/*z* value, H/C, AI_mod_, DBE, and NOSC, respectively. For each FT-ICR MS spectrum, peak intensities of all peaks were normalized by their maximum value to calculate the Bray–Curtis dissimilarity and molecular functional diversity.

The one-way or two-way analysis of variance (ANOVA) was applied to statistically determine differences of compared groups with parameters following a normal distribution at a significance level of *p* < 0.05. Otherwise, the nonparametric Kruskal–Wallis one-way ANOVA was used to examine the difference between compared groups. Statistical analysis was performed using Origin software (version 2024, OriginLab, USA).

## 3 Results and Discussion

### 3.1 Overall Molecular Characteristics of Raw DOM

The hierarchical cluster and heatmap analysis based on the Bay-Curtis dissimilarity of FT-ICR MS peak occurrence and magnitude among all spectra revealed that the clustered subgroup was dependent on the same source and less affected by the eluate drying methods (Figure S1). In addition to the high confidence in the reproducibility of FT-ICR MS spectra, this observation demonstrated that the occurrence and magnitude of FT-ICR MS peaks were more profoundly governed by their sources rather than by the eluate drying methods. A typical Gaussian-like spectral profile was observed in the overall FT-ICR MS spectra and their expanded spectra at each nominal mass for all samples, which was exemplified by the soil DOM eluate in Figures 1, S2, and S3. The elemental composition and molecular classes of all raw DOM eluate tabulated in Tables S1 and S2, respectively, revealed that lignin-like compounds mainly composited by CHO and CHON were the predominated molecules in the DOM with different sources (Figure S2), which was consistent with the results for diverse natural samples.^1,6,34,35,42^ The lignin-like molecules accounted for 83.29% ± 0.21% to 90.03% ± 0.16% of the total magnitudes of the assigned formula, with formula numbers ranging from 5871.67 ± 258.65 to 9651.00 ± 103.12. The CHO molecule number ranged from 4115.00 ± 138.72 in the East Lake water to 8241.33 ± 41.63 in the groundwater, contributing to 71.78% ± 0.93% to 83.70% ± 0.89% of total magnitudes. The CHON molecule number was in the range of 1347.00 ± 77.12 in the tap water to 2841.67 ± 156.53 in the East Lake water, with the magnitude contribution of 7.67% ± 0.01% to 21.68% ± 0.42%, respectively. Although the FT-ICR MS spectra were dominated by singly charged peaks for all samples, considerable numbers of doubly charged peaks were identified in all FT-ICR MS spectra (e.g., the blue formulas in Figures 1, S3, S4, and S5). There were 947.33 ± 107.11, 442.33 ± 45.83, 2045.33 ± 102.57, 1358.67 ± 100.38, and 2353.33 ± 32.56 doubly charged formulas assigned for tap water, East Lake water, soil DOM, Yangtze River water, and groundwater, respectively, accounting for 1.67% ± 0.07% to 8.24% ± 0.47% of total magnitudes. These doubly charged formulas exhibited significantly lower H/C (F value = 1254.9, degree of freedom = 21007, *p* < 0.0001) but significantly higher O/C (F value = 700.3, degree of freedom = 21007, *p* < 0.0001) than those for the singly charged formula in each raw DOM eluate because DOM molecules rich in the carboxylic moiety were more tended to form doubly charged peaks in the ESI(−) treatment due to the deprotonation nature of carboxyl functional groups.^35,43^

**Figure 1:**
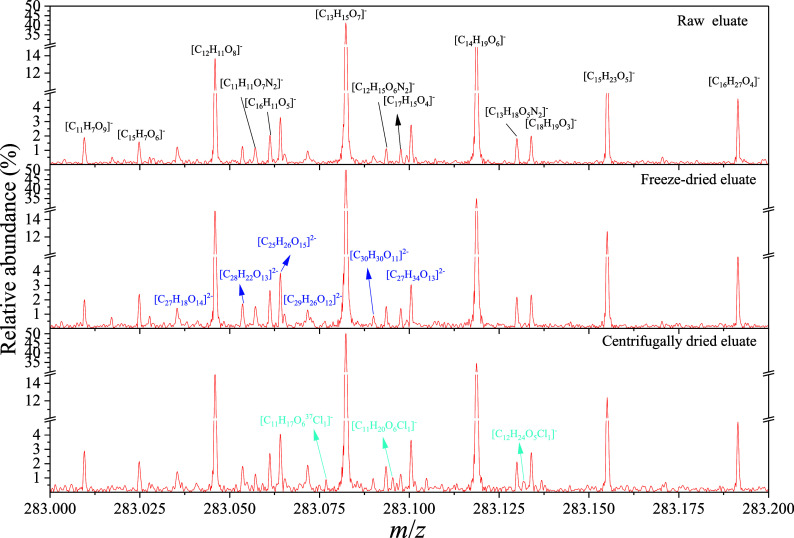
Expanded FT-ICR MS spectra of soil DOM at a nominal mass of 283.

### 3.2 Molecular Properties of DOM Eluate Affected by Drying Methods

The absorbance values at 254 nm in the reconstituted DOM eluate dried by the lyophilization were 40.73% ± 10.24% of these in the raw DOM eluate (Figure S6), suggesting that approximately half of the eluted DOM molecules were lost during the vacuum freeze-drying treatment. By contrast, the vacuum centrifugal concentrator resulted in 10.58% ± 5.86% of DOM molecules lost during the drying treatment in this study. The significant difference (Kruskal–Wallis ANOVA test, *p* = 0.0018) in the DOM recovery between FD-DOM and CD-DOM could be mainly attributed to additional improvement by the centrifugal function in the vacuum centrifugal concentrator used in this study. Therefore, the vacuum centrifugal concentrator is recommended to dry the methanol-based DOM eluate from the viewpoint of DOM recovery.

The higher value of the molecular functional diversity index represents the larger statistically expected difference in the chosen molecular index (e.g., H/C, O/C, AI_mod_, DBE, and NOSC), suggesting higher molecular functional diversity in the examined FT-ICR MS spectrum. Generally, the values of molecular functional diversity indices (e.g., *m*/*z*, DBE, and NOSC) were comparable to these for natural samples reported elsewhere,^40,44,45^ suggesting the similar molecular functional diversity between raw DOM eluate in this study and previous studies. Differing from the significant difference (*p* = 0.0018) in the concentrations of DOM eluate, an insignificant difference was observed in the values of molecular functional diversity indices for each DOM among three treatments (Figure 2, F value = 0.083–1.26, degree of freedom = 29, *p* = 0.16–0.92), suggesting that the FD-DOM and CD-DOM exhibited only minor influences on the overall molecular functional diversity. However, compared to vacuum freeze-drying, vacuum centrifugal drying resulted in lower mean values in the *DF­(m/z)* but higher mean values of *DF­(DBE)* and *DF­(AI*_*mod*_ *)*, indicating the different structural effects of eluate drying methods on DOM molecules.

**Figure 2:**
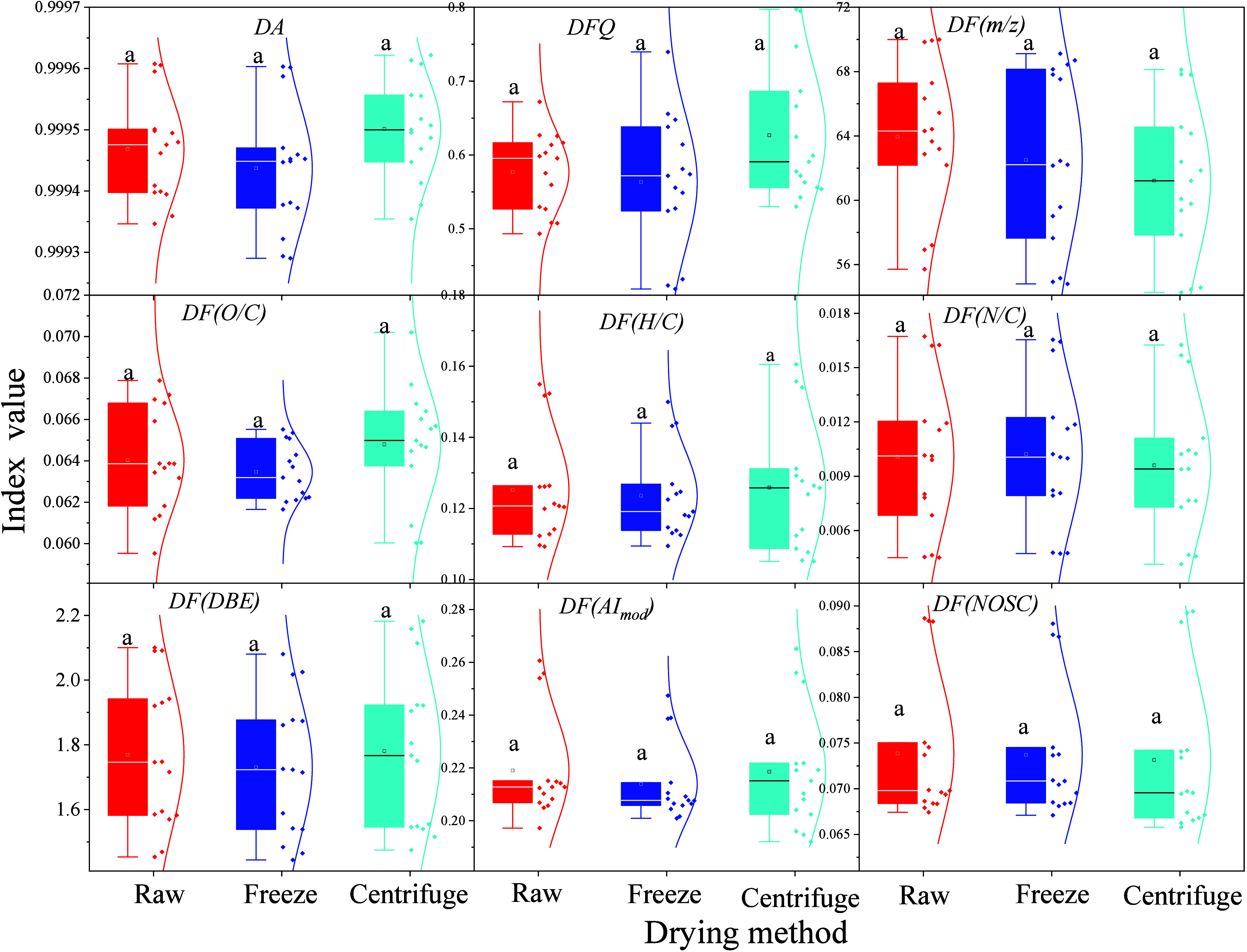
Molecular functional diversity affected by two eluate drying methods. Note, **the identical letter indicates the insignificant difference between the compared groups at** ***p*** > 0.05.

The low-intensity peaks, including doubly charged peaks and peaks with low *S*/*N* values (*S*/*N* ≤ 15), were selected to evaluate the influences of the drying treatment on their occurrence because of the poor reproducibility of low-intensity peaks in the FT-ICR MS spectra.^32,33^ Compared with the raw DOM eluate, the number of assigned formulas with low intensities was insignificantly affected by both drying methods used in this study (ANOVA test: F value = 0.51 *p* = 0.32–0.68, Kruskal–Wallis ANOVA test *p* = 0.73, Figure S7), suggesting that these two methods exhibited negligible influence on the occurrence of low-intensity peaks for DOM eluate with different sources. Similarly, in addition to the labile compounds with H/C ≤ 1.5,^46^ both drying methods had insignificant influence on the assigned formula number of lignin-like compounds, which were the predominant fraction of DOM (Kruskal–Wallis ANOVA test *p* = 0.99, Figure S8). However, the formula numbers of tannin-like compounds and saturated compounds were significantly suppressed by vacuum centrifuge drying (Kruskal–Wallis ANOVA test *p* = 0.014, Figure S8). Meanwhile, vacuum freeze-drying significantly reduced the formula number of saturated compounds. These results suggested that one should be cautious to employ the drying methods used in this study when the saturated compounds were the analytes of interest.

The number of assigned formulas for different elemental compositions affected by drying treatments was illustrated in Figure S9. As tabulated in Table 1, both vacuum freeze-drying and vacuum centrifuge drying methods insignificantly decreased the average number of formulas assigned to CHO and CHON compounds that were dominant in the DOM eluate (Kruskal–Wallis ANOVA test *p* = 0.89 and 0.15, respectively, Figures S2 and S9), which was consistent with the ignorable change in the formula number of lignin-like compounds. Moreover, the DOM drying treatment exhibited a minor influence on the minor fraction of DOM eluate, namely, CHOS and Cl-containing organic molecules (Cl-OM), in this study (Figure S9). For example, the CHOS formula numbers in the FD-DOM and CD-DOM were 96.03% ± 21.05% and 96.50% ± 12.65% of that for the raw DOM eluate (Table 1), respectively. The number of Cl-OM was insignificantly decreased but significantly increased by the vacuum freeze-drying and vacuum centrifuge drying, respectively (Kruskal–Wallis ANOVA test *p* = 0.37 and 0.04, respectively, Figure S9). However, in addition to the contrasting effects, the coefficient of variation (CV) for the formula number ratios of Cl-OM in the raw DOM eluate relative to FD-DOM and CD-DOM was up to 90.03% and 163.97%, respectively, suggesting that the effects of DOM drying on the Cl-OM detection were dependent on their DOM sources.

**Table 1:** Average of Assigned Formula Numbers for Different Elemental Compositions

		Elemental composition					
Sample	Drying method (DOM)	CHO	CHON	CHOS	Cl-OM^a^	Br-OM^b^	Total formula number
Tap water	Raw Elute (Raw DOM)	4312.66 ± 190.59	1347.00 ± 77.12	191.33 ± 30.24	1038.67 ± 51.05	189.67 ± 56.89	7079.33 ± 391.90
	Freeze (FD-DOM)	4125.33 ± 77.15	1332.33 ± 41.67	243.00 ± 4.58	1069.67 ± 21.82	230.67 ± 21.08	7001.00 ± 155.21
	Centrifuge (CD-DOM)	4356.33 ± 128.76	1290.33 ± 38.99	223.00 ± 14.42	1540.67 ± 142.12	197.67 ± 6.66	7608.00 ± 297.56
East Lake	Raw Elute (Raw DOM)	4115.00 ± 138.72	2841.67 ± 156.52	531.33 ± 37.61	81.33 ± 18.01	NA^c^	7569.33 ± 342.26
	Freeze (FD-DOM)	4103.67 ± 37.98	2712.33 ± 46.69	485.00 ± 9.17	170.67 ± 25.77	NA^c^	7471.67 ± 79.25
	Centrifuge (CD-DOM)	3880.33 ± 113.10	2581.00 ± 105.35	463.67 ± 33.00	448.00 ± 184.75	NA^c^	7373.00 ± 64.49
Soil DOM	Raw Elute (Raw DOM)	7136.67 ± 69.82	2442.00 ± 88.50	54.33 ± 1.15	8.00 ± 3.46	NA^c^	9641.00 ± 157.16
	Freeze (FD-DOM)	7029.33 ± 117.01	2414.00 ± 67.67	37.00 ± 1.00	3.33 ± 1.15	NA^c^	9483.67 ± 174.91
	Centrifuge (CD-DOM)	6431.67 ± 271.33	2116.00 ± 79.23	52.33 ± 8.14	321.00 ± 72.51	NA^c^	8921.00 ± 316.74
Yangtze	Raw Elute (Raw DOM)	5827.33 ± 50.90	2660.33 ± 55.19	295.33 ± 16.20	194.00 ± 15.72	2.00 ± 0.00	8979.00 ± 95.39
	Freeze (FD-DOM)	5981.33 ± 129.50	2742.33 ± 113.78	280.00 ± 25.51	118.67 ± 29.87	1.33 ± 1.15	9123.88 ± 232.88
	Centrifuge (CD-DOM)	5685.33 ± 178.00	2410.33 ± 136.55	289.67 ± 17.04	576.67 ± 157.43	NA^c^	8962.00 ± 260.29
Groundwater	Raw Elute (Raw DOM)	8241.33 ± 41.63	2149.67 ± 23.44	391.67 ± 24.09	877.67 ± 77.66	0.67 ± 1.15	11661.00 ± 123.01
	Freeze (FD-DOM)	8691.67 ± 9.61	2386.33 ± 29.77	387.67 ± 0.58	51.67 ± 26.58	NA^c^	11517.33 ± 47.18
	Centrifuge (CD-DOM)	8389.67 ± 601.22	2139.00 ± 192.94	330.00 ± 13.45	984.33 ± 63.71	NA^c^	11843.00 ± 756.73

a Cl-bearing organic matter.

b Br-bearing organic matter.

c Not assigned; values are expressed as the mean ± SD.

### 3.3 Sample-Dependent Effects of DOM Drying Treatment

The intensity-weighted values of typical molecular parameters in the raw DOM eluate, FD-DOM, and CD-DOM from different sources were tabulated in Table S3. Results of two-way ANOVA analysis revealed that no consistent influence of drying methods was observed on the molecular parameters of the DOM eluate from each source. For example, the intensity-weighted *m*/*z* values were insignificantly (F value = 0.68, degree of freedom = 8, *p* = 0.68) affected by the drying treatments in the soil DOM but significantly (F value = 8.91–84.53, degree of freedom = 5 or 8, *p* = < 0.0001–0.04) decreased in DOM from the East Lake, Yangtze River, and groundwater. However, the intensity-weighted NOSC (NOSC_*iw*_) values were insignificantly (F value = 0.35–6.94, degree of freedom = 5, *p* = 0.058–0.58) decreased by the vacuum freeze-drying for the DOM from the soil, Yangtze River, and groundwater but significantly (F value = 27.77 and 201.78, degree of freedom = 5, *p* = 0.0062 and 1.43 × 10^–4^ 0.05) decreased for DOM eluate from the tap water and East Lake. Furthermore, the vacuum centrifuge drying significantly (F value = 18.20 and 46.50, degree of freedom = 5, *p* = 0.0024 and 0.013) decreased the NOSC_*iw*_ values for DOM eluate from the tap water and groundwater. Despite the insignificant effects on the values of bulk molecular parameters (*e.g*., molecular functional diversity indices and formula number of different elemental compositions and molecular classes), the drying treatments used in this study exhibited different influences on DOM eluate from various sources and different molecular parameters for a given DOM source.

An insignificant difference was observed for the formula number of CHO and CHON molecules in most compared groups among different samples and treatments (*p* > 0.05, Figure 3). Similarly to the minor changes in the formula number of CHOS, the formula number ratios of DOM eluate treated by drying were 96.96% ± 4.85% to 100.04% ± 3.78% and 92.67% ± 5.01% to 101.46% ± 5.98% for CHO and CHON molecules, respectively. However, substantial changes caused by drying treatment were observed in the formula number of Cl-OM (*e.g*., chlorinated DBPs) for different DOM samples (Figure 3). For example, the vacuum centrifuge drying increased 48.33%, 450.82%, 3912.50%, and 197.25% number of Cl-OM formulas in the DOM eluate from tap water, East Lake, soil, and Yangtze River, respectively, as compared with the raw DOM eluate, which was visually supported by the exclusively identified [C_11_H_17_O_6_^37^Cl_1_]^−^, [C_11_H_20_O_6_Cl_1_]^−^, and [C_12_H_24_O_5_Cl_1_]^−^ in the centrifugally dried soil DOM (Figure 1). The vacuum freeze-drying profoundly decreased the formula number of Cl-OM molecules in the groundwater and Yangtze River DOM (F value = 14.94 and 303.83, degree of freedom = 5, *p* = < 0.0001 and 0.018), which could be attributed to significant DOM loss during the drying treatment, but increased (F value = 24.22, degree of freedom = 5, *p* < 0.0079) Cl-OM formula number in the East Lake DOM. Moreover, this drying treatment insignificantly increased Cl-OM molecules in the tap water DOM but decreased in the soil DOM (F value = 0.93, degree of freedom = 5, *p* = 0.091). Therefore, we should be discrete to dry DOM eluate if Cl-OM compounds are of critical interest in our research.

**Figure 3:**
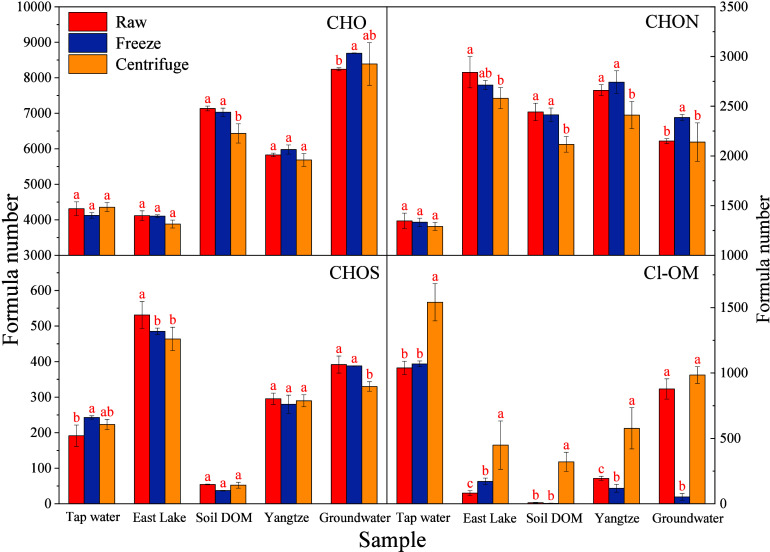
Effects of drying treatments on the chemical formula number of elemental compositions for DOM from different sources. Note, **different red letters indicate the significant difference between compared groups at** ***p*** **< 0.05**.

## 4 Environmental Implication

In this study, the molecular functional diversity indexes at the bulk level were not sensitive to drying treatments using both a vacuum freeze-dryer and a vacuum centrifuge dryer. Moreover, the dominant DOM fractions, including lignin-like compounds and CHO and CHON molecules, were insignificantly affected by drying treatments. These results suggest that the two drying treatments employed can be used to concentrate DOM eluate only if functional diversity indexes are used as molecular proxies for DOM or these DOM fractions are exclusively considered for research purposes. However, these scenarios are rare in ecological and environmental research. In addition to DOM fractions (CHOS molecules and saturated compounds) susceptive to drying treatments, the profoundly affected results of Cl-OM suggest that phosphorus-containing molecules will also be affected by the drying treatment due to the close mass doublet of ^12^C_1_^35^Cl_1_ versus ^16^O_1_^31^P_1_ (Δmass = 0.18 mDa).^34^ These results have highlighted the critical effects of the drying treatment for DOM eluates from disinfected waters,^4,47^ wastewater,^48^ and sediments,^49^ which contain considerable chlorine- and sulfur-containing organic molecules. The vacuum centrifuge drying under low temperatures (*e.g*., ∼ 0 °C) may be suitable for these minor factions of DOM molecules, such as the chlorinated DBPs, which should be further investigated. However, only DOM from five different sources was used in this study. Given the diverse sources of DOM and its substantial differences in molecular classes and elemental compositions among various sources, the different influences of drying treatments on five DOM samples examined in this study have highlighted the necessity of FT-ICR MS-based preinvestigation for the drying treatment of unknown DOM samples. Therefore, the raw DOM eluate diluted with methanol and ultrapure water is strongly recommended for FT-ICR MS measurement without drying treatment, and vacuum centrifuge drying is recommended for DOC measurement to evaluate DOM recovery for the SPE extraction.

## 5 Conclusions

In this study, the effects of typical DOM eluate drying methods on the chemodiversity of DOM from different sources were investigated using FT-ICR MS with all treatments conducted in triplicate. Contrasting with the approximate half loss of DOM by vacuum freeze-drying, vacuum centrifuge drying only resulted in about 10% DOM mass loss. The occurrence and magnitude of FT-ICR MS peaks were more profoundly governed by their sources rather than by the eluate drying methods. The lignin-like compounds mainly assigned to CHO and CHON molecules were the predominant components in DOM from five different sources, with fewer contributions from saturated compounds and CHOS and Cl-OM molecules. Although the overall values of molecular functional diversity indices and the formula number of doubly charged ions and peaks with *S*/*N* ≤ 15 were insignificantly affected by both drying treatments for each DOM among three treatments, the Cl-OM and saturated compounds were significantly affected by drying treatments, particularly for the vacuum centrifuge drying. Therefore, DOM eluate is strongly recommended to be measured by FT-ICR MS only after desired dilution with methanol and/or ultrapure water to minimize the effects of drying treatment on the chemodiversity of DOM from different sources, particularly for the minor DOM fractions such as Cl-DOM and saturated compounds. The results of this study provided valuable evidence for the sample preparation of DOM from various sources for the accurate elucidation of DOM chemodiversity.

## Supplementary Material



## Notes

Molecular functional diversity index equation (**Text S1**), DOM molecular composition (**Table S1**), DOM molecular class (**Table S2**), intensity-weighted molecular parameters (**Table S3**), Bay-Curtis dissimilarity-based hierarchical clustering and heatmap derived (**Figure S1**), van Krevelen diagram (**Figure S2**), FT-ICR MS spectra (**Figures S2**, **S4**, and **S5**), UV absorbance value (**Figure S6**), formula number of doubly charged and low-intensity formulas (**Figure S7**), assigned formula number of different molecular classes (**Figure S8**), and formula number of different elemental compositions (**Figure S9**) (PDF)
