# Supplementary material for: Effects of Eluate Drying on the Chemodiversity of Dissolved Organic Matter Revealed by Ultrahigh-Resolution Mass Spectrometry
Source: ACS Meas Sci Au. 2025 Jun 30;5(4):461–8. doi: 10.1021/acsmeasuresciau.5c00055 (PMC12371599; doi:10.1021/acsmeasuresciau.5c00055)
Supplement: Supplementary file 1 [file tg5c00055_si_001.pdf]

Supporting Information for

Effects of eluate drying on the chemodiversity of dissolved organic matter revealed by ultrahigh-resolution mass spectrometry

Xinyi Chen[a], Qing-Long Fu[a,*], Ziyong Sun[a]

[a] *MOE Key Laboratory of Groundwater Quality and Health, School of Environmental Studies, China University of Geosciences, Wuhan 430074, China*

** Corresponding author: Qing-Long Fu (E-mail address: fuqinglong@cug.edu.cn)*

Summary

Number of pages: 16

Number of text sections: 1

Number of tables: 3

Number of figures: 9

Content

Text S1. Calculation equations for molecular functional diversity index.

Table S1. The molecular composition of different raw DOM eluate.

Table S2. The molecular class of different raw DOM eluates.

Table S3. Intensity-weighted molecular parameters of DOM eluate affected by drying treatment.

Figure S1. The hierarchical clustering and heatmap derived from the Bay-Curtis dissimilarity of all samples. The similarity between samples is indicated as a color gradient from higher similarity (blue) to lower similarity (red). DOM1, DOM2, and DOM3 represented the raw DOM eluate, and DOM eluate dried by vacuum freeze drying and vacuum centrifugal, respectively.

Figure S2. The van Krevelen diagram for singly charged ions and doubly charged ions for raw DOM eluate from different samples.

Figure S3. The broadband FT-ICR MS spectra of soil DOM eluate.

Figure S4. Expanded FT-ICR MS spectra of soil DOM eluate at the nominal mass of 284.

Figure S5. Expanded FT-ICR MS spectra of soil DOM eluate at the *m*/*z* 283.5.

Figure S6. The UV absorbance value at 254 of each DOM eluate. Note, that the eluate was diluted before UV measurement. Note, different red letters indicate the significant difference at $p < 0.05$.

Figure S7. Effects of drying treatment on the formula number of doubly charged formulae and low-intensity formulae. Note, that the identical red letter indicates the insignificant difference at $p > 0.05$.

Figure S8. The assigned formula number of different molecular classes affected by drying treatment. Different red letters indicate the significant difference at $p < 0.05$.

Figure S9. Effects of drying treatment on the formula number of different elemental compositions. Note, different red letters indicate the significant difference at $p < 0.05$.

Text S1. Calculation equations for molecular functional diversity index.

In this study, an in-house MATLAB script code was employed to quantify the conceptually different diversity indices, namely, abundance-based Gini-Simpson index *DA* (Eq.(1)) and modified Rao quadratic entropy index *DF* (Equation (2)) [1, 2].

$$D_A = 1 - \sum_{i=1}^{N} p_i^2 \qquad (2)$$

where *N* is the total number of molecular formulae in the spectrum, and p_i is the relative signal intensity of molecular formula *i*. The range of *DA* value is 0 to 1, representing the probability of two randomly selected molecular formulae that are different. The greater the *DA* value suggests the higher the diversity. When the distribution is uniform (p_i=1/*N*), *DA* reaches its maximum value of 1-1/*N*.

$$D_F = \sum_{i=1}^{N-1} \sum_{j=i+1}^{N} p_i \times p_j \times \left| c_i - c_j \right| \qquad (2)$$

where: *DF* reflects the difference of the theoretically expected value of the selected parameters between any two molecular formulae in a spectrum. c_i is the value of a specific property parameter (*m*/*z* value, H/C, AI_{mod}, DBE, and NOSC) of the given molecular formula *i*.

The comprehensive functional diversity was quantified with the Equation (3):

$$DFQ = \sum_{i=1}^{N-1} \sum_{j=i+1}^{N} p_i \times p_j \times \frac{\sum_{k=1}^{n} \left| c_{ik} - c_{jk} \right|}{n} \qquad (3)$$

where: *DFQ* reflects the difference of the theoretically expected value of the selected parameters between any two molecular formulae in a spectrum. c_{ik} c_{ik} is the value of a specific property parameter *k* (*m*/*z* value, H/C, AI_{mod}, DBE, and NOSC) of the given spectra *i*.

Table S1. The molecular composition of different raw DOM eluate.

Elemental composition	Tap Water		East Lake		Soil DOM		Yangtze River		Groundwater	
	Intensity (%)	Count	Intensity (%)	Count	Intensity (%)	Count	Intensity (%)	Count	Intensity (%)	Count
CHO	75.82 ±2.38	4312.67 ±190.59	71.78 ±0.93	4115.00 ±138.72	83.90 ±0.06	7136.67 ±69.82	79.86 ±0.41	5827.33 ±50.90	83.70 ±0.89	8241.33 ±41.63
CHON	9.97 ±0.10	1347.00 ±77.12	21.68 ±0.42	2841.67 ±156.53	14.56 ±0.26	2442.00 ±88.50	16.16 ±0.38	2660.33 ±55.19	7.67 ±0.01	2149.67 ±23.44
CHOS	1.92 ±0.80	191.33 ±30.24	5.09± 0.55	531.33 ±37.61	1.40± 0.23	54.33 ±1.15	2.05 ±0.29	295.33 ±16.20	1.72 ±0.17	391.67 ±24.09
CHOP	0.64 ±0.10	104.00 ±5.57	0.00 ±0.00	0.00 ±0.00	0.00 ±0.00	0.00 ±0.00	0.01 ±0.01	3.00 ±1.73	0.13 ±0.02	24.00 ±1.73
CHONS	0.23 ±0.05	34.67 ±5.77	0.73 ±0.06	198.67 ±27.43	0.09 ±0.02	7.33 ±1.15	0.24 ±0.01	61.33 ±11.15	0.45 ±0.09	144.67 ±18.61
CHONP	0.19 ±0.09	30.33 ±12.66	0.09 ±0.00	14.00 ±1.00	0.01 ±0.01	0.67 ±1.15	0.10 ±0.02	13.00 ±2.65	0.26 ±0.02	80.33 ±6.03
CHOSP	0.04 ±0.01	9.67 ±1.53	0.00 ±0.00	0.00 ±0.00	0.00 ±0.00	1.33 ±1.53	0.04 ±0.03	7.33 ±4.16	0.03 ±0.01	10.67 ±1.53
Cl-bearing	9.91 ±2.02	1038.67 ±51.05	0.63 ±0.15	81.33 ±18.01	0.04 ±0.03	8.00 ±3.46	1.53 ±0.17	194.00 ±15.72	6.04 ±0.75	877.67 ±77.66
Br-bearing	1.20 ±0.39	189.67 ±56.89	0.00 ±0.00	0.00 ±0.00	0.00 ±0.00	0.00 ±0.00	0.01 ±0.00	2.00 ±0.00	0.00 ±0.00	0.67 ±1.15
Cl+Br	0.07 ±0.01	9.33 ±1.15	0.00 ±0.00	0.00 ±0.00	0.00 ±0.00	0.00 ±0.00	0.00 ±0.00	0.00 ±0.00	0.02 ±0.01	5.00 ±2.65

Note: values are expressed as the mean ± SD.

Table S2. The molecular class of different raw DOM eluate.

Molecular class	Tap Water		East Lake		Soil DOM		Yangtze River		Groundwater	
	Intensity (%)	Count	Intensity (%)	Count	Intensity (%)	Count	Intensity (%)	Count	Intensity (%)	Count
Carbohydrate	0.42 ±0.10	74.67 ±17.10	0.11 ±0.01	18.67 ±3.21	0.07 ±0.01	20.33 ±1.53	0.22 ±0.01	33.67 ±1.53	0.04 ±0.02	10.67 ±4.04
Aminosugars	0.18 ±0.03	32.00 ±9.17	0.18 ±0.02	36.33 ±3.51	0.21 ±0.02	52.33 ±3.79	0.29 ±0.04	57.67 ±5.69	0.05 ±0.01	20.00 ±4.36
N-bearing saturated compounds	0.24 ±0.07	49.67 ±6.43	0.48 ±0.04	101.33 ±13.32	0.74 ±0.06	169.33 ±15.82	0.42 ±0.09	105.00 ±18.19	0.24 ±0.01	111.00 ±4.58
N-free saturated compounds	5.20 ±0.60	419.33 ±30.53	8.24 ±0.66	667.33 ±25.70	8.77 ±0.35	887.67 ±12.50	5.54 ±0.77	646.67 ±38.21	4.98 ±0.44	937.00 ±73.61
Tannins	4.75 ±0.51	568.67 ±82.71	1.89 ±0.27	227.67 ±25.50	3.82 ±0.08	505.67 ±8.62	2.98 ±0.92	432.67 ±76.81	3.16 ±0.18	623.67 ±27.39
Lignins	87.26 ±0.42	5871.67 ±258.65	86.35 ±0.87	6489.00 ±303.40	83.29 ±0.21	7512.67 ±112.19	87.08 ±0.23	7384.33 ±51.96	90.03 ±0.16	9651.00 ±103.12
Unsaturated Hydrocarbons	0.01 ±0.01	1.33 ±1.15	0.00 ±0.00	0.00 ±0.00	0.00 ±0.00	0.67 ±0.58	0.01 ±0.00	2.00 ±0.00	0.00 ±0.00	0.67 ±0.58
Condensed Aromatic Structures	0.74 ±0.04	154.00 ±6.24	0.22 ±0.03	72.67 ±12.06	2.21 ±0.02	402.67 ±15.70	0.89 ±0.17	229.33 ±38.53	1.28 ±0.13	525.33 ±30.44
Others	1.19 ±0.06	96.00 ±19.47	2.54± 0.06	169.00 ±1.73	0.89 ±0.03	99.00 ± 3.61	2.57 ±0.14	172.33 ±6.03	0.22 ±0.02	46.33 ±4.62
MLB_L	9.33 ±0.36	810.33 ±74.45	14.68 ±0.78	1179.33 ±52.54	12.52 ±0.24	1380.67 ±25.32	11.11 ±1.20	1189.67 ±68.41	6.94 ±0.53	1324.67 ±77.93

Note: values are expressed as the mean ± SD.

Table S3. Intensity-weighted molecular parameters of DOM eluate affected by drying treatment.

Sample	Drying	Molecular characteristics									
		Measure *m/z*	H/C	O/C ($\times10^{-1}$)	N/C ($\times10^{-3}$)	S/C ($\times10^{-3}$)	Cl/C ($\times10^{-3}$)	X/C	AI_{mod} ($\times10^{-1}$)	DBE	NOSC ($\times10^{-1}$)
Tap water	Raw	400.06±9.72^{a}	1.25±0.00^{a}	4.89±0.01^{a}	8.24±0.7^{a}	1.60±0.55^{a}	9.07±1.05^{a}	1.26±0.00^{a}	2.43±0.01^{a}	8.30±0.13^{a}	-2.32±0.03^{a}
	Freeze	386.64±1.43^{b}	1.27±0.00^{b}	4.70±0.02^{b}	8.80±0.15^{a}	2.02±0.34^{a}	9.90±0.29^{a}	1.28±0.00^{b}	2.38±0.01^{ab}	7.97±0.04^{b}	-2.87±0.06^{b}
	Vacuum	392.8±5.19^{ab}	1.28±0.01^{b}	4.57±0.07^{c}	8.15±0.25^{a}	1.67±0.33^{a}	14.97±1.30^{b}	1.30±0.01^{c}	2.34±0.05^{b}	7.97±0.03^{b}	-3.25±0.23^{c}
East Lake	Raw	402.58±2.45^{a}	1.31±0.00^{a}	4.55±0.02^{a}	19.7±0.43^{a}	3.88±0.37^{ab}	0.69±0.14^{a}	1.31±0.01^{a}	2.22±0.02^{a}	7.95±0.09^{a}	-3.3±0.08^{a}
	Freeze	381.71±1.99^{b}	1.32±0.01^{b}	4.37±0.04^{b}	19.38±0.44^{a}	4.15±0.63^{a}	1.55±0.40^{a}	1.33±0.01^{a}	2.22±0.02^{a}	7.59±0.10^{b}	-3.82±0.15^{b}
	Vacuum	395.22±1.40^{c}	1.31±0.01^{a}	4.50±0.07^{a}	18.71±0.68^{a}	2.98±0.35^{b}	4.48±2.47^{b}	1.31±0.01^{a}	2.23±0.04^{a}	7.82±0.14^{a}	-3.4±0.26^{a}
Soil DOM	Raw	410.7±1.13^{a}	1.20±0.00^{ab}	4.75±0.01^{a}	11.24±0.17^{a}	1.00±0.14^{ab}	0.04±0.02^{a}	1.20±0.00^{a}	2.87±0.01^{a}	9.47±0.03^{ab}	-2.13±0.06^{a}
	Freeze	406.32±4.56^{a}	1.18±0.01^{b}	4.78±0.05^{a}	11.27±0.15^{a}	0.61±0.40^{b}	0.02±0.00^{a}	1.18±0.00^{a}	2.94±0.02^{b}	9.60±0.07^{a}	-1.93±0.16^{a}
	Vacuum	409.83±9.86^{a}	1.21±0.01^{a}	4.64±0.07^{b}	10.76±0.56^{a}	1.30±0.21^{a}	2.01±0.76^{b}	1.22±0.01^{b}	2.82±0.04^{a}	9.33±0.12^{b}	-2.50±0.28^{a}
Yangtze River	Raw	413.82±5.10^{a}	1.25±0.02^{a}	4.71±0.09^{a}	13.44±0.33^{a}	1.62±0.20^{a}	1.61±0.16^{a}	1.25±0.02^{a}	2.54±0.05^{a}	8.82±0.15^{a}	-2.63±0.34^{a}
	Freeze	404.69±1.41^{b}	1.25±0.00^{a}	4.65±0.02^{ab}	13.51±0.37^{a}	1.50±0.19^{a}	1.19±0.26^{a}	1.25±0.00^{ab}	2.56±0.01^{a}	8.76±0.01^{ab}	-2.75±0.06^{a}
	Vacuum	406.28±3.77^{b}	1.27±0.01^{a}	4.55±0.09^{b}	11.89±0.56^{b}	2.05±0.39^{a}	4.80±1.61^{b}	1.27±0.01^{b}	2.48±0.04^{a}	8.56±0.10^{b}	-3.17±0.30^{a}
Groundwater	Raw	466.27±3.41^{a}	1.19±0.01^{a}	4.73±0.03^{a}	4.86±0.07^{a}	1.32±0.08^{a}	3.04±0.35^{a}	1.19±0.01^{a}	2.79±0.03^{a}	10.34±0.12^{a}	-2.25±0.12^{a}
	Freeze	448.36±1.92^{b}	1.17±0.01^{a}	4.80±0.03^{a}	5.06±0.03^{a}	1.04±0.08^{a}	0.09±0.05^{b}	1.17±0.01^{b}	2.90±0.03^{b}	10.36±0.08^{a}	-1.98±0.13^{a}
	Vacuum	452.31±5.2^{b}	1.23±0.01^{b}	4.44±0.12^{b}	4.75±0.29^{a}	1.89±0.22^{b}	3.74±0.49^{a}	1.23±0.01^{c}	2.69±0.04^{c}	9.93±0.11^{b}	-3.21±0.37^{b}

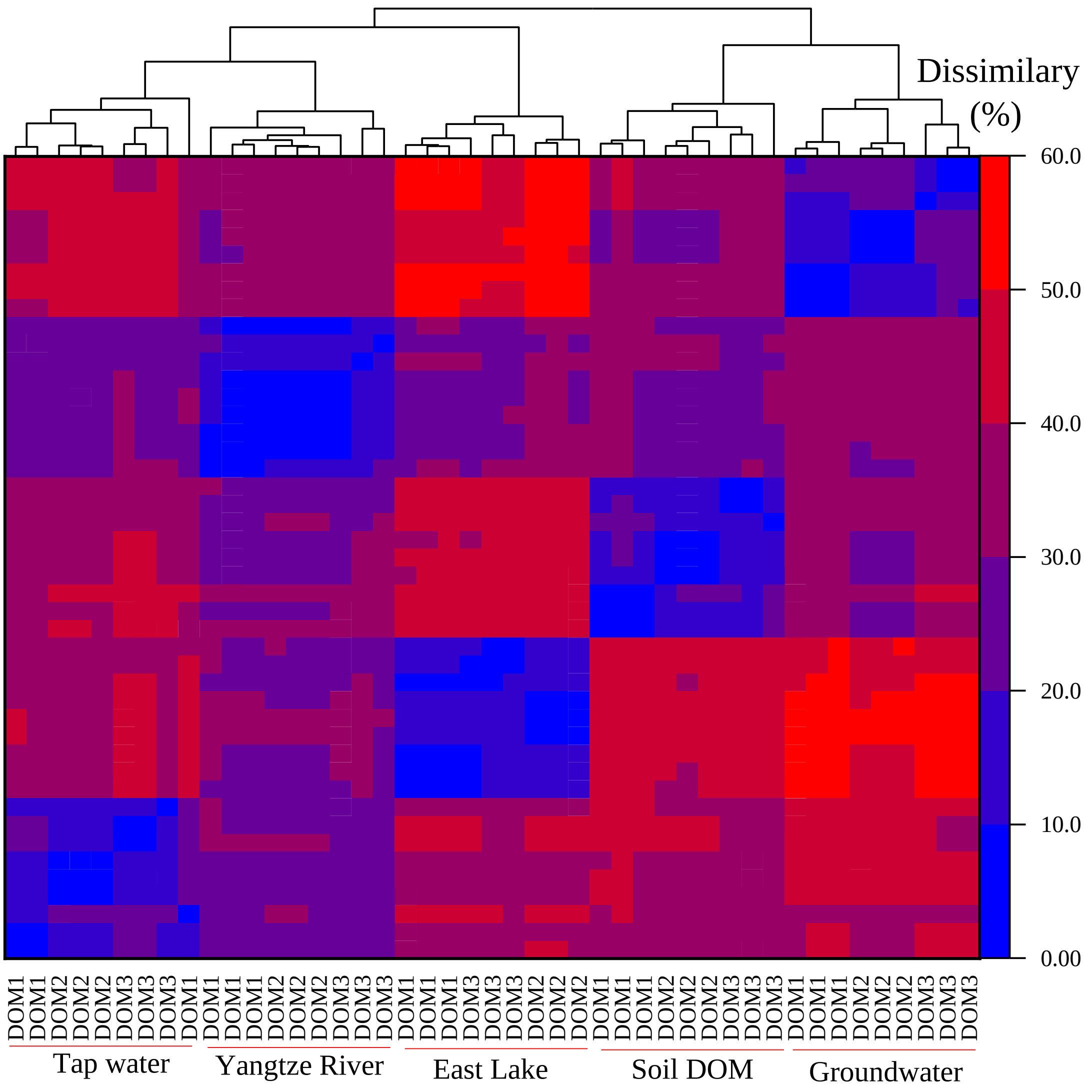

Figure S1. The hierarchical clustering and heatmap derived from the Bay-Curtis dissimilarity of all samples. The similarity between samples is indicated as a color gradient from higher similarity (blue) to lower similarity (red). DOM1, DOM2, and DOM3 represented the raw DOM eluate, and DOM eluate dried by vacuum freeze drying and vacuum centrifugal, respectively.

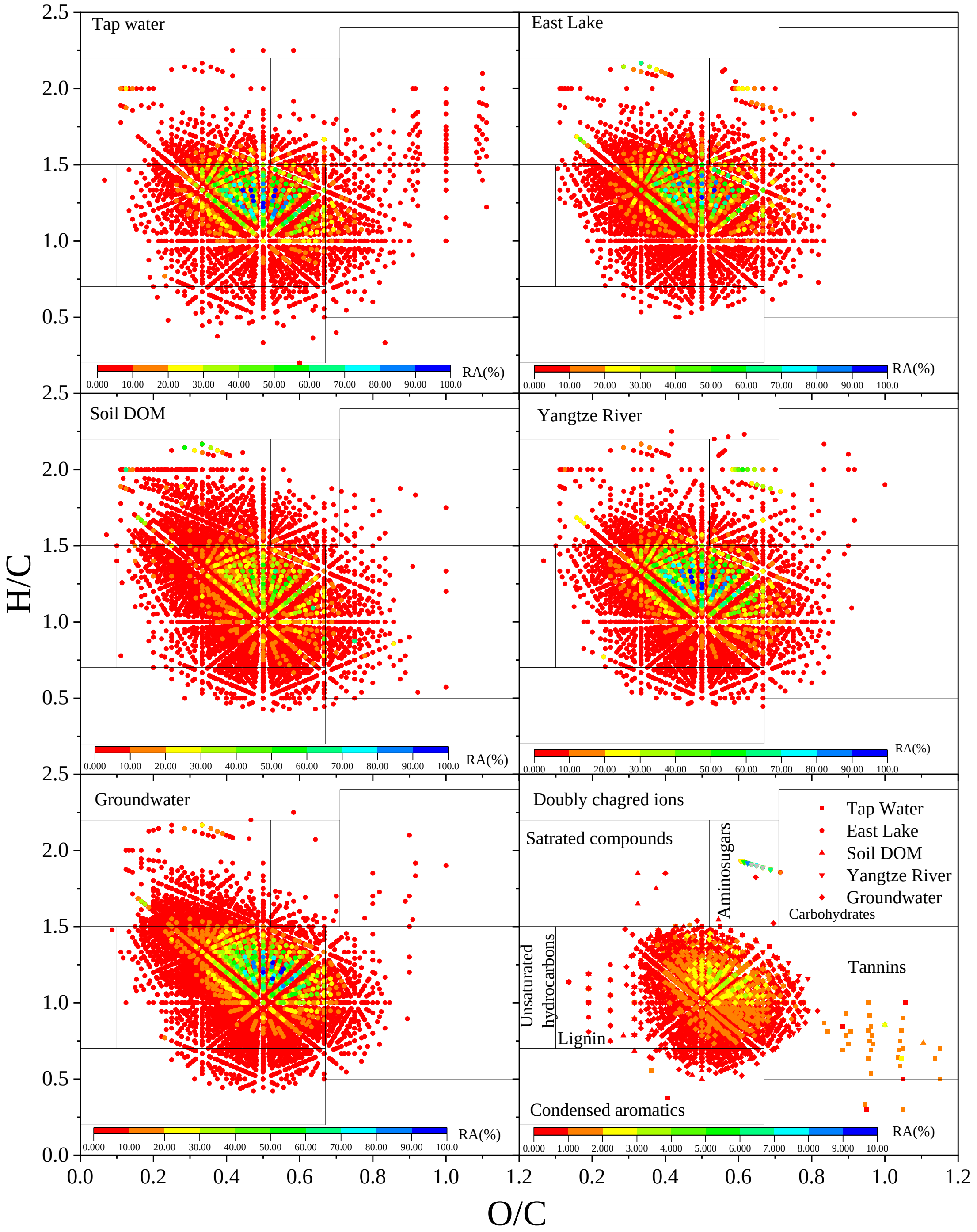

Figure S2. The van Krevelen diagram for singly charged ions and doubly charged ions for raw DOM eluate from different samples.

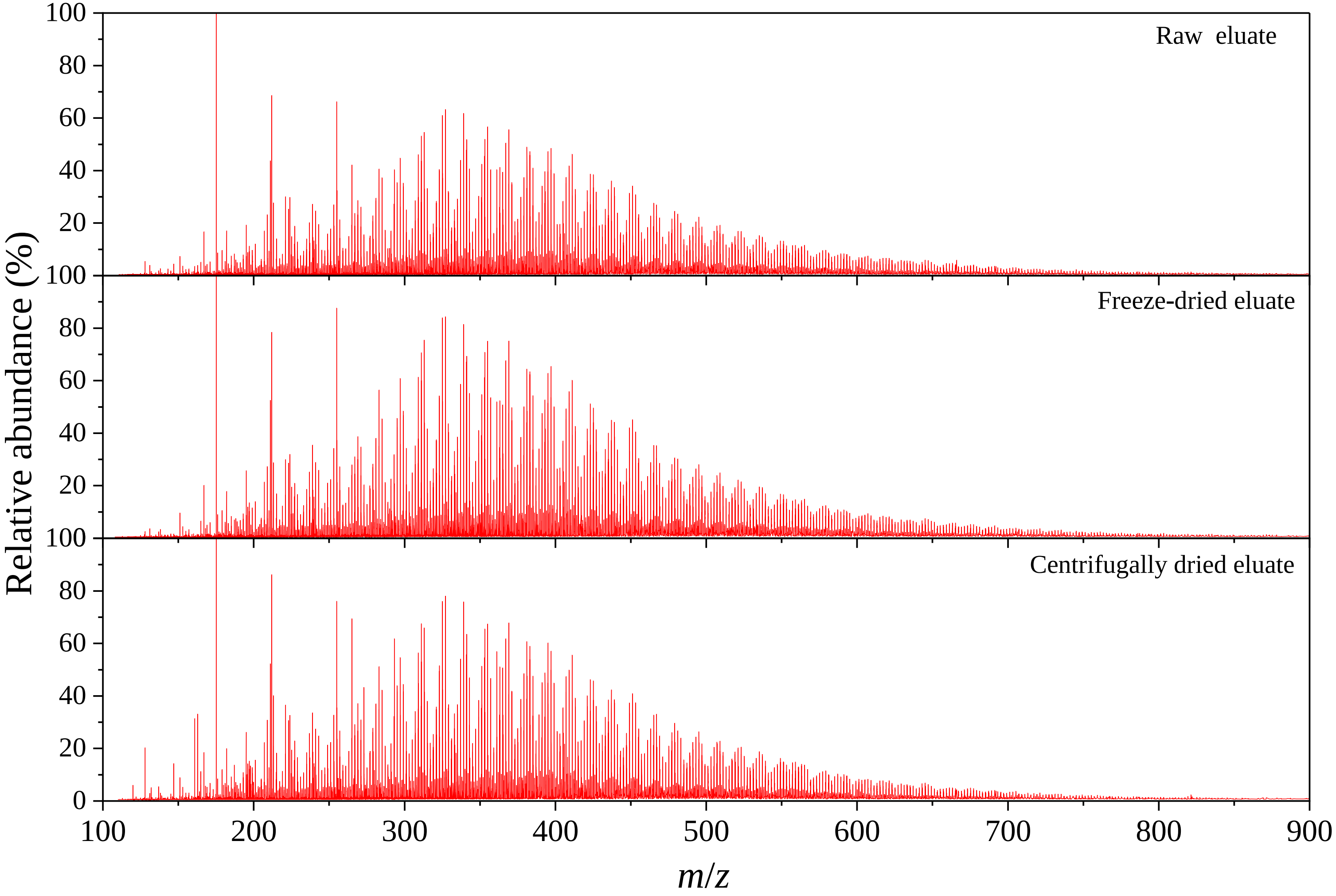

Figure S3. The broadband FT-ICR MS spectra of soil DOM eluate.

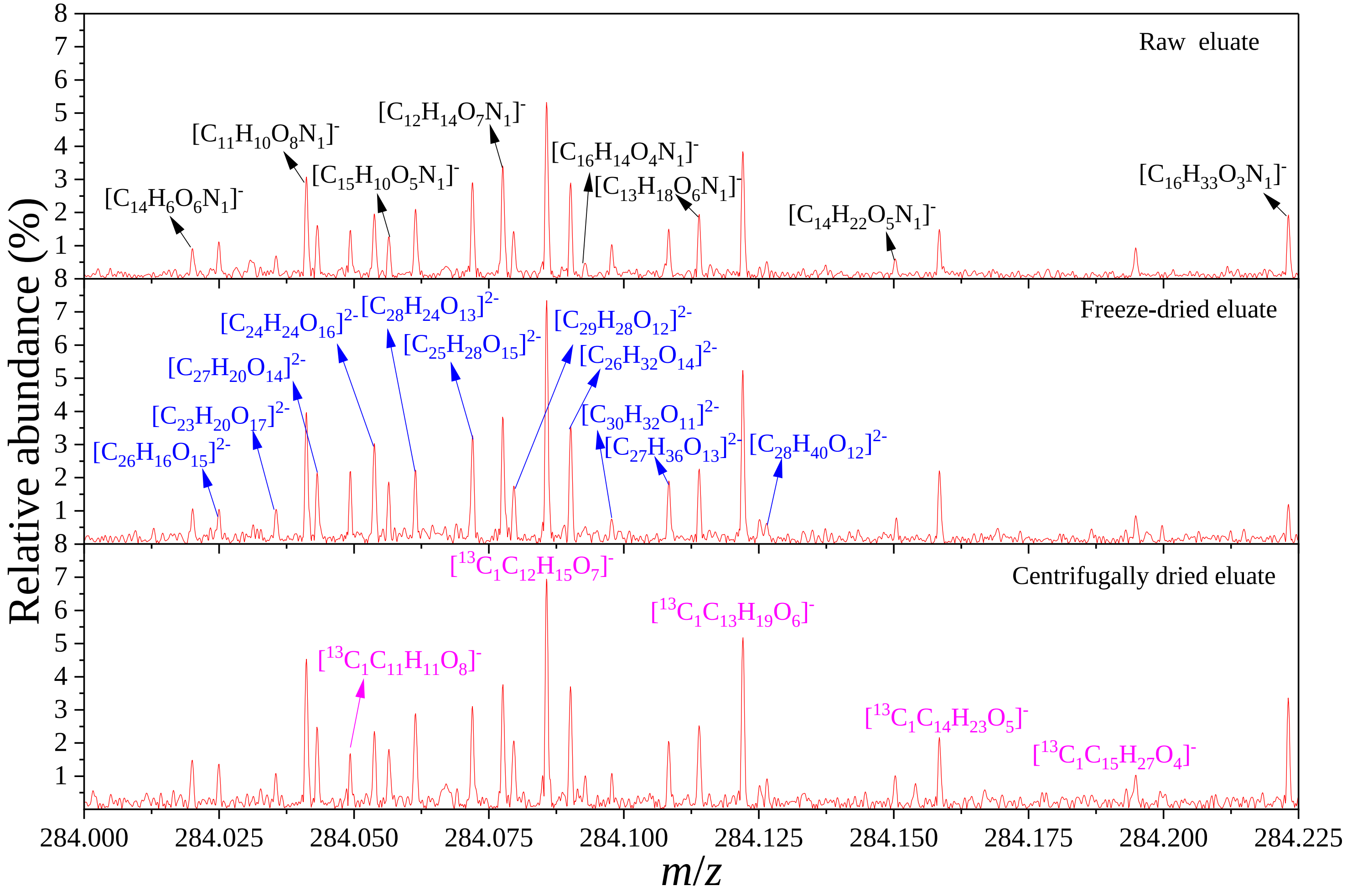

Figure S4. Expanded FT-ICR MS spectra of soil DOM eluate at the nominal mass of 284.

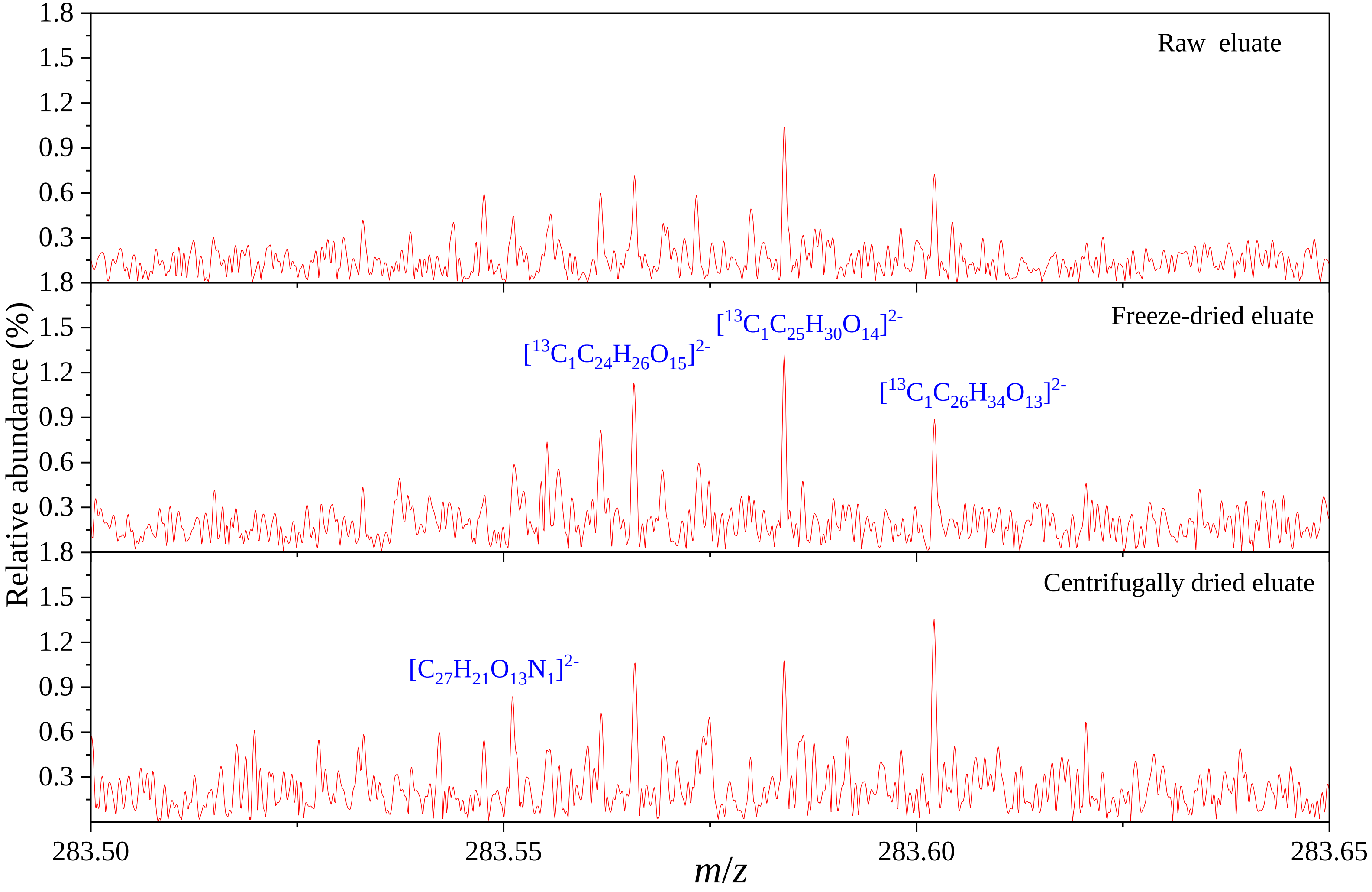

Figure S5. Expanded FT-ICR MS spectra of soil DOM eluate at the *m*/*z* 283.5.

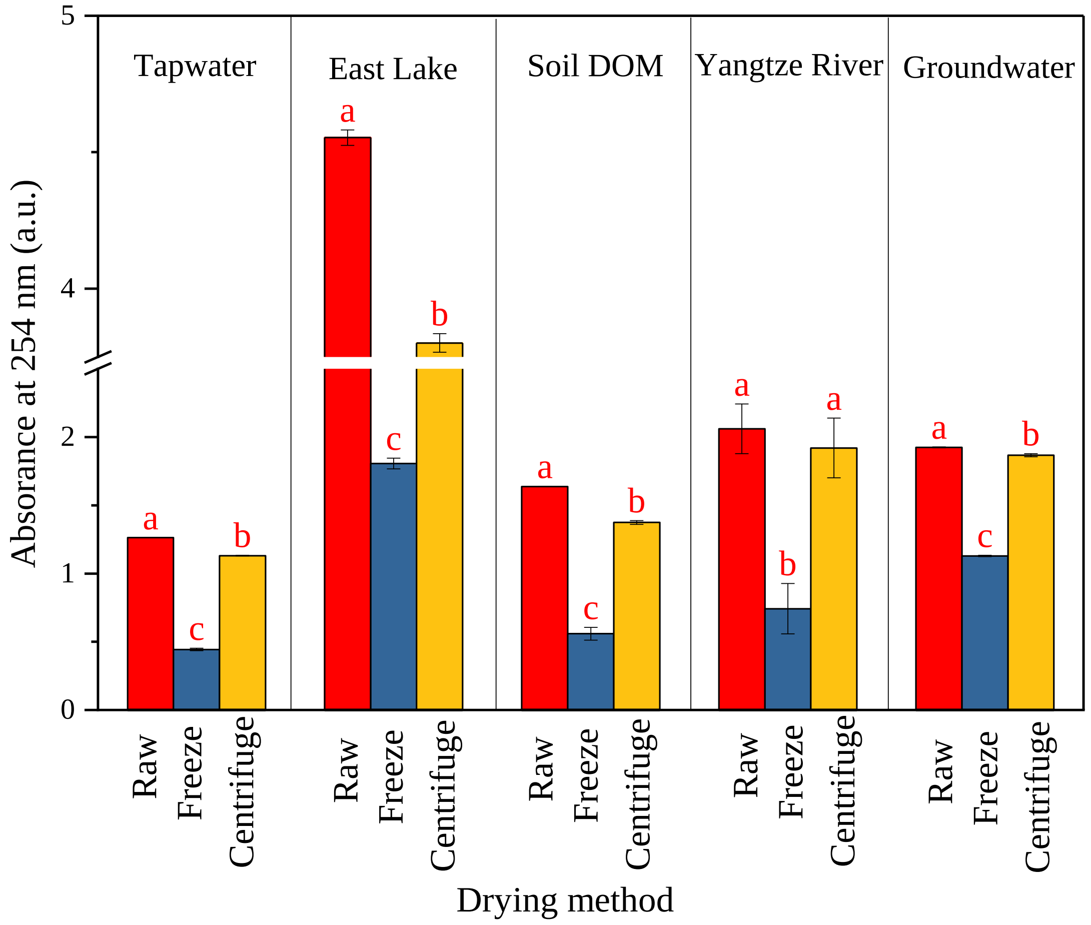

Figure S6. The UV absorbance value at 254 of each DOM eluate. Note, that the eluate was diluted before UV measurement. Note, different red letters indicate the significant difference at p <0.05.

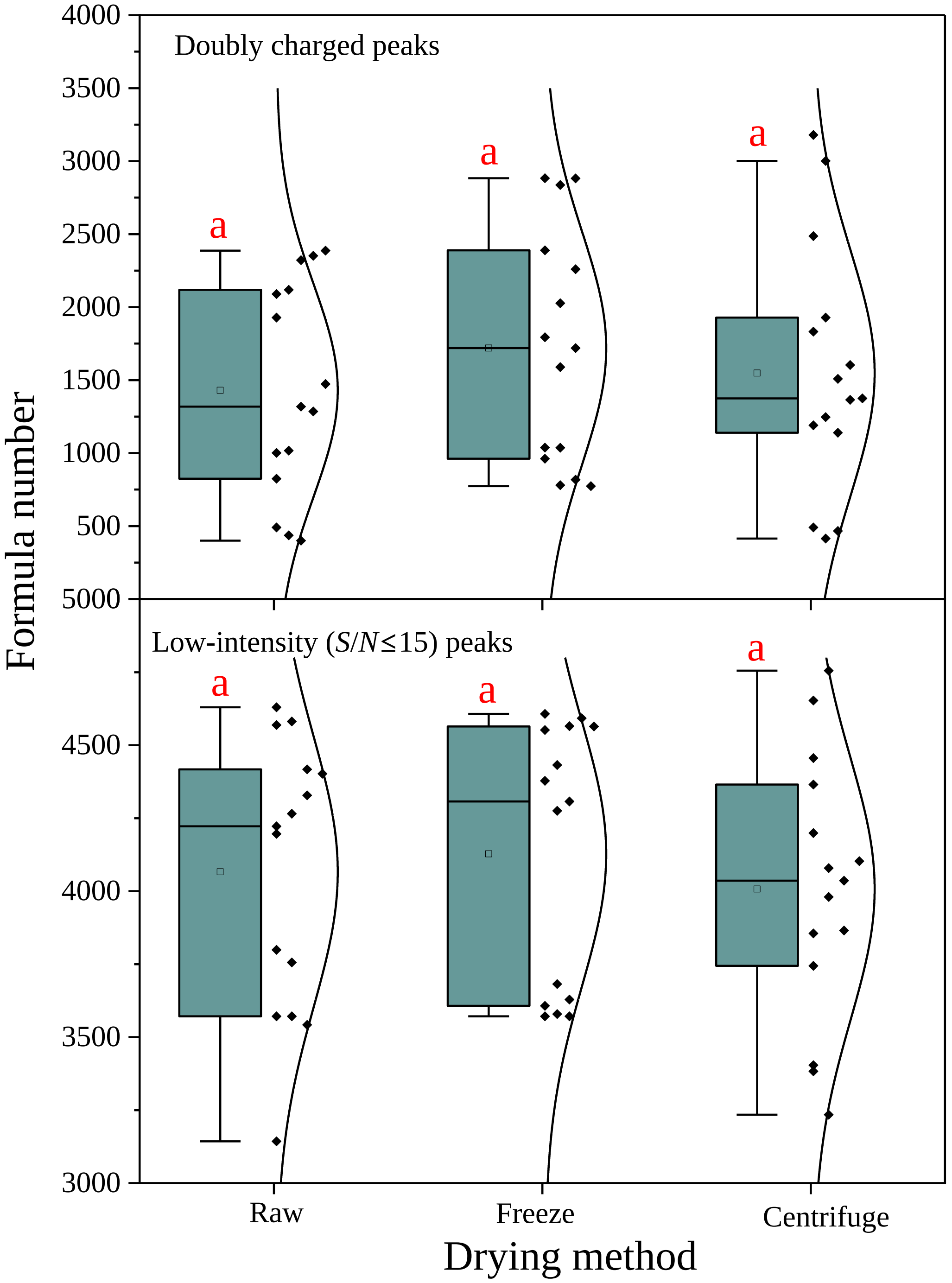

Figure S7. Effects of drying treatment on the formula number of doubly charged formulae and low-intensity formulae. Note, that the identical red letter indicates the insignificant difference at p >0.05.

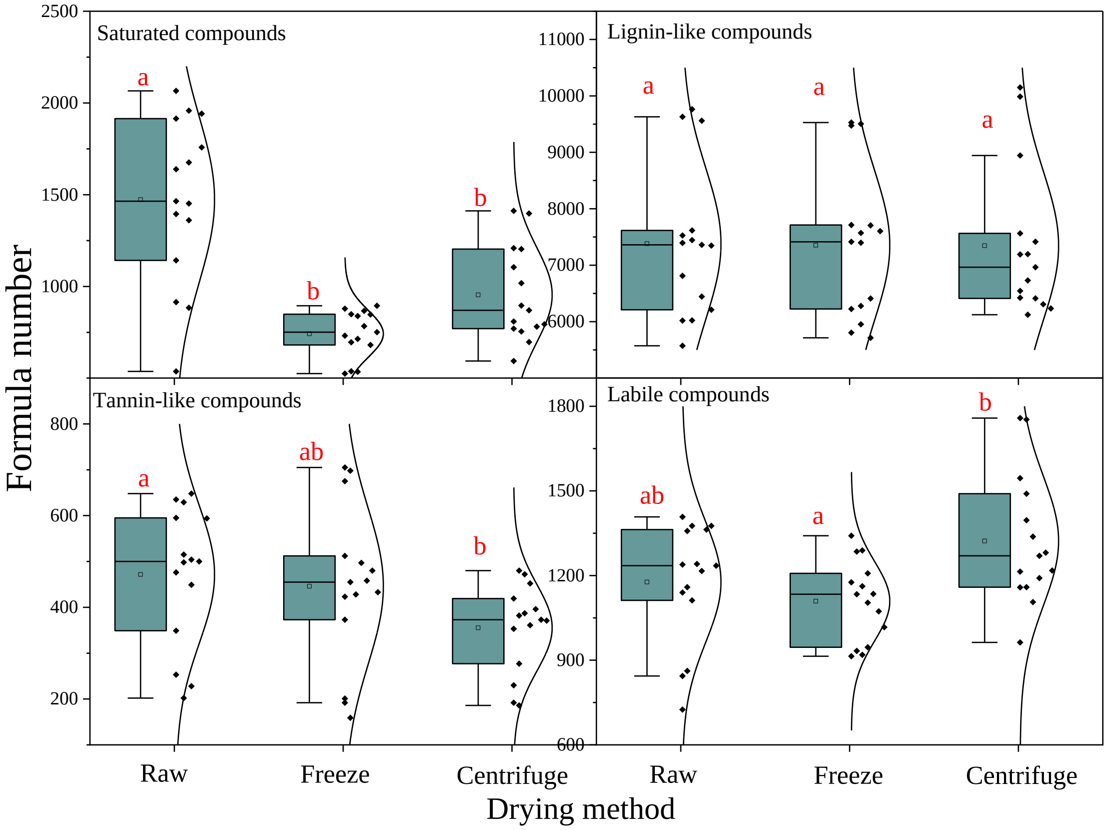

Figure S8. The assigned formula number of different molecular classes affected by drying treatment. Different red letters indicate the significant difference at $p < 0.05$.

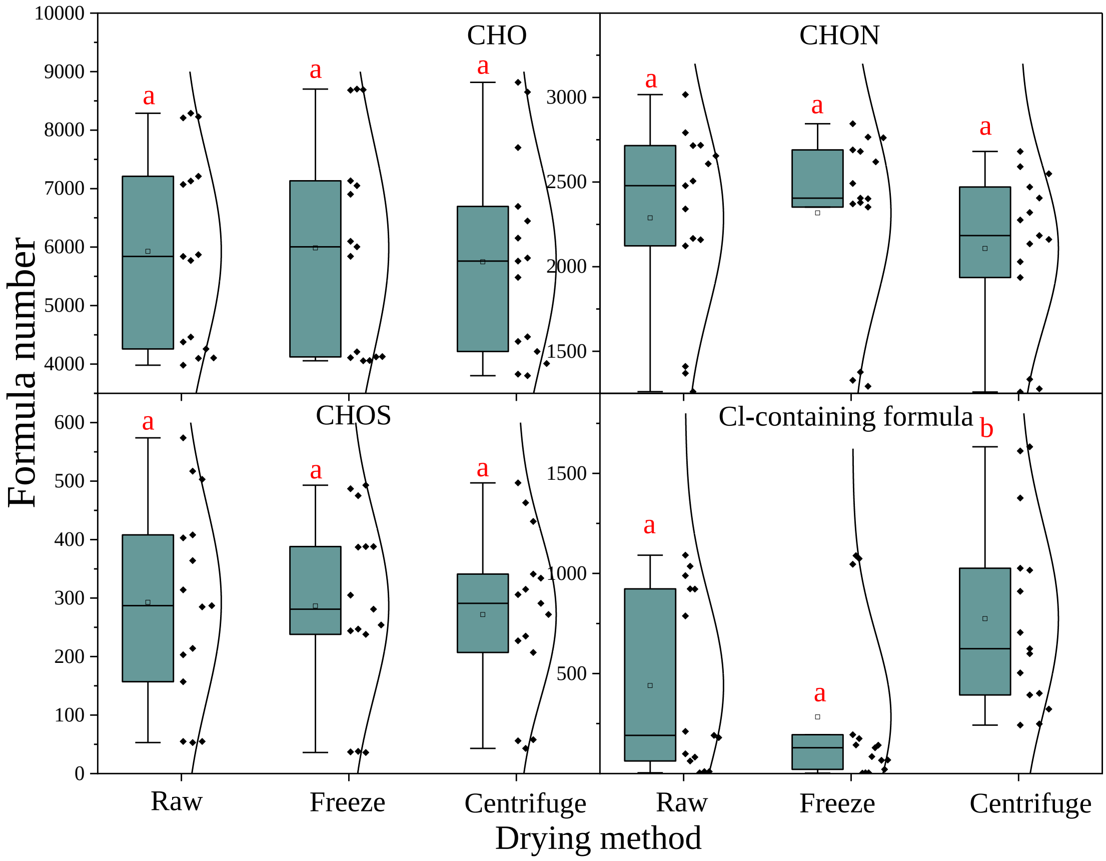

Figure S9. Effects of drying treatment on the formula number of different elemental compositions. Note, different red letters indicate the significant difference at p <0.05.

Reference:

(1) Botta-Dukát, Z. Rao's quadratic entropy as a measure of functional diversity based on multiple traits. *J. Veg. Sci.* **2005**, *16* (5), 533-540.

(2) Mentges, A.; Feenders, C.; Seibt, M.; Blasius, B.; Dittmar, T. Functional Molecular Diversity of Marine Dissolved Organic Matter Is Reduced during Degradation. *Front. Mar. Sci.* **2017**, *4*, 194. DOI: 10.3389/fmars.2017.00194.
